# Supplementary material for: Using Laser-Doppler Flowmetry to Evaluate the Therapeutic Response in Dentin Hypersensitivity
Source: Int J Environ Res Public Health. 2020 Nov 26;17(23):8787. doi: 10.3390/ijerph17238787 (PMC7731012; doi:10.3390/ijerph17238787)
Supplement: Supplementary file 1 [file ijerph-17-08787-s001.pdf]

Table S1. Recorded dental blood flow values.

Nr.	Experimental Condition	Tooth Nr.	Initially		Immediately		After 24 h		After 7 days		After 1 month	
			Blood Flow Mean Value (PU)	Standard Deviation σ	Blood Flow Mean Value (PU)	Standard Deviation σ	Blood Flow Mean Value (PU)	Standard Deviation σ	Blood Flow Mean Value (PU)	Standard Deviation σ	Blood Flow Mean Value (PU)	Standard Deviation σ
1	**Laser Nd:YAG + gel**	41	6.2	1.2	9.6	2.3	8.2	1.5	3.4	0.7	2.6	0.6
2		42	7.2	1.0	9.2	1.7	5.5	1.0	2.7	0.6	2.4	0.6
3		43	5.4	0.9	8.6	1.3	4.7	0,7	2.4	0.4	2.3	0.7
4		11	4.2	0.8	6.5	1.1	4.7	0.9	4.6	0.8	3.6	0.7
5		12	6.8	1.1	7.8	1.2	6.0	1.2	4.1	0.8	3.6	0.6
6		13	5.9	1.0	6.8	1.2	6.3	1.1	3.1	0.7	3.9	0,6
7		13	6.3	1.6	11.8	2.2	8,7	1.6	2.2	1.7	3.4	0.9
8		23	4.8	0.9	9.3	2.1	6.2	1.4	2.7	0.5	3.2	0.6
9		13	5.3	1.2	9.8	1.8	8.7	1.5	3.2	1.0	3.4	0.9
10		11	4.6	0.9	7.3	1.3	6.2	1.4	3.4	0.8	3.2	0.6
11		13	9.5	1.6	11.2	1.9	9.7	1.6	5.7	1.0	6.0	1.0
12		12	11.5	2.2	10.8	1.7	9.5	1.6	6.2	1.1	6.8	1.4
13		11	12.0	1.9	13.7	2.4	11.5	2.1	8.8	1.6	8.5	1.6
14		13	16.9	2.7	18.2	3.0	16.5	2.7	8.2	1.5	9.6	1.9
15		12	16.8	2.5	17.4	2.8	15.4	2.4	7.4	1.1	8.2	1.4
16		11	15.1	2.5	16.9	2.7	15.2	2.5	10.1	2.0	11.0	2.1
17		12	7.2	1.2	9.4	2.3	8.2	1.5	4.4	0.7	4.5	0.7
18		11	9.2	1.7	7.4	1.4	5.5	1.0	3.7	0.7	4.4	0.9
19	**Gel**	31	7.5	1.2	7.9	1.1	4.8	0.7	4.1	0.8	4.8	1.0
20		32	6.0	0.9	6.8	1.3	5.7	0.8	4.8	0.9	4.2	0.8
21		33	6.0	0.8	6.2	1.0	5.2	0.8	4.9	0.9	4.8	0.9
22		21	4.0	0.7	5.0	0.8	5.7	1.0	3.8	0.8	3.2	0.7
23		22	3.9	0.7	4.5	0.8	4.2	0.8	3.8	0.7	3.7	1.0
24		23	7.2	1.1	8.4	1.1	8.7	1.3	6.4	1.1	5.4	1.0
25		43	7.3	1.3	8.9	1.5	6.3	1.0	4.3	0.8	5.0	0.9
26		33	9.4	1.4	7.7	1.2	4.5	0.8	3.8	0.8	5.6	1.1

27	21	6.3	1.2	8.0	1.3	5.3	0.9	5.0	0.9	4.3	0.8
28	23	6.4	1.4	7.9	1.2	5.5	1.0	4.8	0.9	4.6	0.8
29	21	12.9	2.2	11.0	2.1	11.5	2.2	8.5	1.5	10.5	1.9
30	22	8.4	1.4	8.3	1.4	6.8	1.2	5.7	0.9	6.2	1.1
31	23	9.3	1.4	9.5	1.6	6.8	1.3	5.1	0.8	5.7	1.0
32	21	15.1	2.2	14.9	2.1	14.5	2.1	12.4	1.9	11.2	1.8
33	22	15.9	2.5	15.2	2.4	13.7	2.3	10.2	2.0	10.8	2.0
34	23	16.5	2.7	15.9	2.4	14.2	2.3	9.7	2.0	11.0	2.1
35	21	8.5	1.5	7.4	1.2	5.8	0.9	4.1	0.8	6.2	1.0
36	22	6.1	0.9	5.8	0.8	5.7	0.8	4.8	0.7	5.2	0.8
